# Ethical issues in implementation research: a discussion of the problems in achieving informed consent

**DOI:** 10.1186/1748-5908-3-52

**Published:** 2008-12-17

**Authors:** Jane L Hutton, Martin P Eccles, Jeremy M Grimshaw

**Affiliations:** 1 Department of Statistics, University of Warwick, Coventry, CV4 7AL, UK; 2 Institute of Health and Society, University of Newcastle, Newcastle upon Tyne, NE2 4AA, UK; 3 Clinical Epidemiology Programme, Ottawa Health Research Institute, Ottawa, Canada

## Abstract

**Background:**

Improved quality of care is a policy objective of health care systems around the world. Implementation research is the scientific study of methods to promote the systematic uptake of clinical research findings into routine clinical practice, and hence to reduce inappropriate care. It includes the study of influences on healthcare professionals' behaviour and methods to enable them to use research findings more effectively. Cluster randomized trials represent the optimal design for evaluating the effectiveness of implementation strategies. Various codes of medical ethics, such as the Nuremberg Code and the Declaration of Helsinki inform medical research, but their relevance to cluster randomised trials in implementation research is unclear. This paper discusses the applicability of various ethical codes to obtaining consent in cluster trials in implementation research.

**Discussion:**

The appropriate application of biomedical codes to implementation research is not obvious. Discussion of the nature and practice of informed consent in implementation research cluster trials must consider the levels at which consent can be sought, and for what purpose it can be sought. The level at which an intervention is delivered can render the idea of patient level consent meaningless. Careful consideration of the ownership of information, and rights of access to and exploitation of data is required. For health care professionals and organizations, there is a balance between clinical freedom and responsibility to participate in research.

**Summary:**

While ethical justification for clinical trials relies heavily on individual consent, for implementation research aspects of distributive justice, economics, and political philosophy underlie the debate. Societies may need to trade off decisions on the choice between individualized consent and valid implementation research. We suggest that social sciences codes could usefully inform the consideration of implementation research by members of Research Ethics Committees.

## Background

Improved quality of care is a policy objective of health care systems around the world. Research findings are sometimes implemented by helping professionals to acquire skills or knowledge, sometimes by making systems changes within health care organisations, and sometimes by legislation which restricts or controls practice. Over the past decade, health care systems have invested heavily in the development of clinical practice guidelines and associated quality improvement interventions [1]. However, these efforts have had variable success [2].

Implementation research is the scientific study of methods to promote the systematic uptake of clinical research findings into routine clinical practice, and hence to reduce inappropriate care [3]. It includes the study of influences on the behaviour of health-care professionals and health care organisations. The emphasis is generally on how treatments can be delivered effectively, rather than on the measuring the difference an idealised treatment makes. Experimental studies that use cluster randomised designs are generally more appropriate for evaluating interventions in implementation research than individual patient randomised controlled trials [4]. Cluster randomized trials randomize an intact social unit (cluster) to an intervention and collect data from individuals within that social unit. In implementation research, a cluster may be defined as an individual health care professional, a family practice, a hospital department, or a hospital, and data are commonly collected on patients cared for in the cluster. Cluster randomized trials are commonly undertaken to minimize the risk of contamination that could occur in a patient randomized trial, if the care of control patients was influenced by the experience of the health care professional providing care to experimental patients [4]. Various codes of medical ethics, such as the Nuremberg Code [5] (see text below) and the Declaration of Helsinki [6] inform medical research. We have previously examined their applicability to cluster randomized trials in general [7], but their application to cluster randomised trials in implementation research is not obvious.

### Key ethical considerations: The Nuremberg code

*1. Voluntary consent of the human subject is absolutely essential. Ascertaining the quality of the consent rests upon each individual: responsibility which may not be delegated*.

*2. The experiment should ... yield fruitful results for the good of society*.

*3. Anticipated results must be justified by background knowledge*.

*4. Avoid all unnecessary physical and mental suffering and injury*.

*5. Not conducted if a priori reason to believe death or disability will occur*.

*6. Degree of risk taken to be balanced by the humanitarian importance*.

*7. Proper preparations should be made to protect the experimental subject*.

*8. Only conducted by scientifically qualified persons*.

*9. Subject should be at liberty to end the experiment*.

*10. Early stopping of experiment if risk of injury, disability, death*.

The primary ethical requirement of consent (central to statistical and biomedical codes of conduct [8-10]) raises particular issues for cluster randomised designs [7,11]. Examples of three cluster randomized trials in implementation research are described in the text below.

### The NEXUS Trial [12]

*This study evaluated the effectiveness of audit and feedback and educational reminder messages to implement the UK Royal College of Radiologists' guidelines for lumber spine and knee x-ray in UK general practices. The study was undertaken in six radiology departments and the 247 general practices that they served. The study design was a before-and-after pragmatic cluster randomised controlled trial using a 2 × 2 factorial design. A randomly chosen subset of general practice patient records (paper and computerised) were examined to assess concordance with criteria derived from the guidelines. The effect of educational reminder messages (expressed as x-ray requests per 1,000 patients) was an absolute change of -1.53 (95% CI: -2.5, -0.57) lumbar spine requests and of 1.61 (95% CI: -2.6, -0.62) knee x-ray requests, relative reductions of approximately 20%. Similarly, the effect of audit and feedback was an absolute change of -0.07 (95% CI: -1.3, 0.9) lumbar spine x-rays requests and an absolute change of -0.04 (95% CI: -0.95, 1.03) for knee x-rays requests, relative reductions of about 1%. None of the differences in concordance between groups were statistically significant*.

### The COGENT (Computerised Guidelines Evaluation in the North of England) Trial [13]

*This was a before-and-after cluster randomised controlled trial, which used a two by two incomplete block design to evaluate the use of computerised decision support (CDSS) to implement clinical guidelines for the primary care management of two conditions: asthma in adults and angina. Practices eligible to participate in the study were those with one of two computing systems, and with at least 50% of the general practitioners reporting use of their practice computer system to view clinical data and for acute prescribing. Process of care data were collected in two ways: by electronic retrieval from the computerised medical record and by abstraction from paper medical records. (At the time of the study the majority of general practices had both electronic and paper records on the same patient.) Patient-based outcomes were assessed by postal surveys using a range of generic and condition specific measures administered at three points in time: approximately a year before the intervention; just before the intervention and approximately a year after the intervention. There were no significant effects of CDSS on consultation rates, process of care measures (including prescribing) or any quality of life domain for either condition. Levels of use of the CDSS were low*.

### The DREAM Trial [14]

*This was an evaluation of the effectiveness and efficiency of an area-wide 'extended' computerised diabetes register, which incorporated a full structured recall and management system, actively involved patients, and included clinical management prompts to primary care clinicians based on locally-adapted evidence based guidelines. The trial, in 58 general practices in three Primary Care Trusts in the northeast of England, was a pragmatic cluster randomised controlled trial with the general practice as the unit of randomisation. The computerised structured recall and management system improved care for people with diabetes. Patients in intervention practices were more likely to have at least one diabetes appointment recorded (OR 2.00, 95% CI 1.02, 3.91), to have a recording of a foot check (OR 1.87, 95% CI 1.09, 3.21), have a recording of receiving dietary advice (OR 2.77, 95% CI 1.22, 6.29), and have a recording of blood pressure (BP) (OR 2.14, 95% CI 1.06, 4.36). There was no difference in mean HbA1c or BP levels, but the mean cholesterol level in patients from intervention practices was significantly lower (-0.15 mmol/l, 95% CI -0.25, -0.06). There were no differences in patient-reported outcomes, or in patient-reported use of drugs or uptake of health services. NHS investigation and treatment costs, and costs to patients were not significantly increased by the intervention; there were administrative costs and there may have been an impact of the intervention on costs within general practice*.

The conduct of cluster randomized trials in implementation research raise a series of questions: What is consent, and who should give it? What does the freedom to withdraw from an experiment mean in implementation research? Indeed, should implementation research be considered 'biomedical research' for ethical purposes? Although the use of medical records in research is considered by the Council for International Organizations of Medical Sciences [15], these guidelines claim that public health and other forms of health care research designed to contribute directly to the health of individuals or communities can be distinguished from biomedical research. The Declaration of Helsinki [6] on biomedical research states that a 'research protocol should always contain a statement of the ethical considerations involved ...'. However, the ten ethical considerations, listed in the text below, are not easily translated into the context of implementation research. While ethical justification for randomised controlled trials relies heavily on the current state of clinical knowledge and individual consent, for implementation research aspects of distributive justice, economics and political philosophy inform the debate, and the ethical theories of virtue, duty, and utility are important. In this paper, we discuss the ethical challenges relating to consent in cluster trials in implementation research.

## Discussion

### Requirement for consent

Is consent necessary for cluster randomized trials in implementation research? Seeking consent poses potential problems, such as causing bias or distress [16,17]. In the case of behaviour change, interventions, knowledge of the intervention, or of the existence of a trial could affect the outcome behaviour. For example, knowing implementation of guidelines was the aim of a trial might encourage professionals to study those guidelines and thus bias the study results. This risk of bias is recognized in the Declaration of Helsinki (point 26) as a legitimate scientific reason why one might chose not to seek consent [6]. Further, providing information particularly under the Council of the International Organisation of Medical Sciences recommendations to provide information on 26 separate items [15] represents a considerable administrative task, so that it might be attractive to avoid consent.

It may also be legitimate to consider whether consent is necessary if the risks to the individual patient are minimal [16,18]. It can be argued that the distinction between implementation research and service development is blurred, often small, and that the requirement for ethics approval may hinder the development of implementation science. Many service developments that could legitimately be the subject of implementation evaluations are conducted in service settings without any consideration of consent for practitioner or patient. A Medline search from 1966 to 2006 on 'natural experiment' in article titles identified 19 reports of health evaluations. A non-systematic sample (the three most recent and therefore most likely to reflect current practice) found no mention of ethical approval [19-21]. An additional consideration is that the risks to individual patients are likely to be small if implementation is based on rigorous evidence of clinical effectiveness, and if the normal ethical duty of the professional to do the best for their patient overrides any study requirements. In contrast, there may be some risks to a health care professional if they are found to be practicing suboptimally.

Our experiences in implementation research in the early 1990s suggested that some ethics committees were confused about this. On at least one occasion, an ethics committee argued that a cluster randomized implementation research trial did not need to be formally considered by an ethics committee. However, as implementation research does incur potential risks for patients and health care professionals, and as implementation researchers might not be well-placed to judge these risks, we believe that formal ethics review should be required. A review of a cluster randomized trial in implementation research should decide whether the quality of the design and the study team are adequate [10], as well as the scope and timing of consent. Indeed, one might argue that service developments should be subject to some form of ethical review, given that they pose not dissimilar risks.

### Scope and timing of consent

In a patient randomized trial of a treatment, patients are asked whether they are willing to consent to randomization to the treatment or control, and willing to participate in all other aspects of a trial, such as regular review visits with their physician, or completion of outcome questionnaires. For cluster randomised trials in implementation research, it is useful to make distinctions that are often not explicit in ordinary randomised controlled trials of treatments. In particular, we consider consent to randomization and consent for other aspects of the trial at the level of the cluster and patient separately.

### Seeking consent for randomization to intervention and control arms

#### Health care providers and organisations

Consent might be sought from those who are the focus of an intervention. Professionals will usually be the primary subjects, and one might seek consent for an intervention intended to affect them. However, if a health care professional chooses not to participate in a study, they are in effect denying their patients the potential benefits of participation. Health care providers ought to do the best for their patients, and have an obligation to improve their skills and knowledge. This might suggest that they should have a high threshold for opting out of implementation research studies. Opting out could be based on the belief that there are significant risks for their patients, or that the opportunity costs of participation are substantial and will be to the detriment of the care of other patients. We (MPE, JMG) have used different approaches to consenting health care professionals, often for pragmatic reasons. In the NEXUS trial [12], we successfully argued that the trial interventions were the equivalent of low risk service developments and that the requirement to seek consent from all potential healthcare professionals may make the project unfeasible or bias our assessment of the study outcome. As a result, we informed all general practitioners within the study areas that there was an ongoing trial but did not explicitly seek their consent. When the interventions were rolled out, we received fewer than five complaints from over 1,000 general practitioners involved in the study. Within the COGENT trial [13], we sought consent from one representative doctor within each participating general practice in the belief that, in this matter, they represented their whole practice.

#### Patients

The level at which an intervention is delivered may determine whether patients can opt in or out [7]. For interventions delivered at the level of the health care professional, it is unclear whether one could ever reasonably seek consent for randomization to intervention and control arms from individual patients who may be affected by the trial interventions. This can render the idea of consent meaningless [7]. It is unclear how far along the chain of responsibilities consent should or can be sought. For example, a trial to evaluate whether an educational intervention would improve district nurses' treatment of leg ulcers might randomise nurses to receive the intervention, or not. Nurses could be recruited and consented. If more than one district nurse served an area, patients could be asked whether they preferred a participating or non-participating nurse: although the intervention is not given to the patients, they might have preferences. In contrast, evaluation of an intervention to improve outpatient care might require involvement by hospital managers, consultants, and junior hospital staff. One could argue that consent should be sought from all junior staff – but should this then extend to all general practitioners who might refer their patients to be seen in outpatients? If so, then should the consent of all the patients who might be referred be sought? Whom one requires to consent will considerably affect the logistics of a trial. The further along the chain one goes, the less feasible seeking consent becomes. For example, the NEXUS trial [12] attempted to reduce inappropriate lumbar spine x-ray requests; potentially this could affect any patient with current or future low back pain. It is not possible to seek consent at the time of randomization from patients who may present with low back pain in the future. While it might be possible to seek consent from patients when they present with low back pain, it is unclear what consent is being sought. Their general practitioner will already have received the intervention. If a patient decided that they did not want to receive care influenced by the intervention, how can the general practitioner minimize the influence of the intervention for that individual patient? If individual patients in the control arm wished to receive care influenced by the intervention, they would have to change to a general practitioner in the intervention arm. Such change would contaminate the randomization. We argue from our experience that it is often nonsensical to seek consent from individual patients to randomization to intervention and control arms. The obligation on professionals, as stipulated by professional bodies such as the General Medical Council, to do their best for their patients overrides any study obligations and should protect patients from inappropriate care. Patients are not generally regarded as having a responsibility to enter randomised controlled trials [22], although some people regard permitting routine health data to be used in research as the duty of a citizen [23].

### Seeking consent for data collection and other aspects of study conduct

Consent might be sought for the use of routinely held data or for the collection of additional data, with or without invasive procedures. For patients who are not directly the subjects of an intervention, one might seek consent to extract data from their medical records [15,24]. Consent might be sought at the point at which records are used, or before any records are used. Not asking consent for access to records minimises impact on patients, and has been advocated [11]. For routine information, it is possible to access data without peoples' knowledge or consent, both to minimise the impact on patients (Declaration of Helsinki, point 21) and to enhance the validity of data [25]. However, the value of simple information and courtesy to research subjects should not be overlooked [16,26]. In the DREAM trial [14], patients had already consented, or were being consented, to their data being held within the existing diabetes register. The study involved no extra 'routine' data collection, and the data extracted for the trial evaluation were anonymised before being sent for analysis; all data held for analysis was held in accordance with the Data Protection Act. For the patient-based questionnaire study, the investigators sought additional patient consent to complete one survey at the time of sending the first questionnaire. The three Local Research Ethics Committees (LRECs) covering the study sites approved the trial on this basis, although one LREC required 'opt-out' patient consent to be sought for participation in the trial. Their stated reason was that, although patients were explicitly consented to be on the register, the consent letter they had signed had not specified 'research' as a use to which their information could be put. The investigators therefore sent an 'opt out' consent letter to all patients on the register in that primary care trust site; 477 out of 4577 (10.4%) patients invited to participate opted out of the trial.

Studies might require subjects to opt-in, or allow them to opt-out. The potential implications for study feasibility of requiring individual patient consent for data collection may be substantial. A quarter of total research funds were spent on consent processes in one study that achieved only 50% participation [23]. We believe that the decision about whether individual patient consent for data collection should be based on considerations of both risk and feasibility. If patients are being contacted to complete a postal survey, then they can clearly be explicitly ask to complete a consent form (*e.g*., COGENT used this method) and thus be consented during this process. Return of a survey implies implicit consent to provide data, but in order to post a questionnaire, access to clinical data is needed to judge eligibility, and a contact address is required. The patient's clinician could be responsible for this, but it is a substantial additional task. For use of routine administrative data in a tightly controlled research environment across a large population, individual patient consent might be waived [24].

#### Proxy consent and coercion

Patients are sometimes deemed incompetent and other people, designated proxies, are allowed to give consent on their behalf. A proxy is usually the next of kin or a legally authorised representative [24]. Proxy consent for professionals cannot coherently be based on incompetence, but might be pragmatically necessary for both professionals and patients. There are many possible proxies who might give consent: consultant committees, medical directors, chief executives, individual doctors, or local research ethics committees. Despite proxy consent, there may still be problems in a situation where there are hierarchies of proxies. Ethics committees may allow access to patient records, but general practitioners may subsequently insist on individual patient consent before allowing access, from their own concerns for patient privacy or their perceived liability. This lengthens and slows any study [27]. Thus, proxy consent can easily misfire: well intentioned over-protective proxies go beyond the law, and threaten public health and epidemiology [25,27-29]. Proxy consent can also have associated inducements or penalties. A senior person or committee might decide that an implementation research trial will include a group of professionals, with disadvantages for those who do not participate, such as loss of some desired status. Participation might earn credits for post-graduate education, which ensures additional funds for individual doctors, providing an inducement. Inducements might coerce doctors into, for example, increasing the uptake of vaccinations or screening. This can lead to patients' being put under pressure. There are restrictions on inducements offered to patients to participate in randomised controlled trials, and to doctors to enroll patients. Any degree of coercion of doctors sits oddly alongside these restrictions. In a patient randomised trial, a patient's refusal should not be allowed to damage the doctor-patient relationship. Ideally, this principle would extend to doctor-doctor or doctor-manager relationships. Coerced participants in implementation research might well produce false results, and so research such participants is likely to be a waste of resources, hence unethical. Alternatively, one might argue that there will always be some healthcare professionals and patients who will always resist change and resent enforced education, and that research that includes resentful participants is therefore usefully pragmatic.

#### Introducing bias by seeking consent

One might rule that individual informed consent must always be obtained. Several studies have assessed the impact of such laws on participation rates and representation. Studies in family practice (USA) or general practice (UK) settings reported similar active consent rates for researchers to have access to patients' medical records: 67% and 61% respectively [30,31]. Of those not consenting, the balance between active refusal and non-response rates differed: 25% and 8% in the USA, compared with 7% and 32% in UK. Worryingly, the USA study showed significant differences in important clinical characteristics between the three subgroups. This at least raises the possibility that study participants would not be representative, thereby limiting the inferences that could be drawn from the study. Other studies have also demonstrated this phenomenon. In a randomised trial of 'opt-in' versus 'opt-out' strategies for an observational study of angina that required clinic attendance, response was defined as attendance at a clinic [32]. The 'opt-in' response rate was 38%, compared to 50% in the 'opt-out' group; the latter group were in poorer health. The potential social, scientific, and financial costs of seeking consent are demonstrated by the hospital-based study in Canada that spent a quarter of the budget to achieve only 50.6% participation in a disease register [23]. This study reported substantial differences between those who consented and those who did not, such that no general scientific conclusions based on the consenters would be valid. In the DREAM Trial [14], 10% of patients declined to participate. Because the research team had no access to these patients' data, the impact of their exclusion is unknown. Considering this evidence, if individual consent is required, then implementation research that requires access to medical records should be abandoned because, even with considerable expenditure on consent, the results of studies are very likely to be substantially biased and misleading. A possible solution could be for patients, when they join a practice, to give a general consent to direct or indirect participation in implementation research that need not be renewed for each new implementation research study [16].

### Which ethics?

#### Ethical theories

There are several important general theories that provide ways in which ethical questions can be approached [33]: Aristotelian virtue ethics, Kantian-type deontological ethics, and consequentialist ethics, or closely related utilitarian ethics. A consequentialist ethic would balance the implication of the informed consent requirement (that implementation research cannot provide valid results, and hence opportunities to improve people's health are lost) with the consequences of not requiring informed consent. The utilitarian approach would assign probabilities and values to the various outcomes, such as misleading results or infringement of autonomy, and reach a semi-quantitative rule. One could justify the informed consent requirement by a Kantian-type duty: the duty of the researcher to seek consent is absolute and takes priority over any duty to act so that knowledge may be gained and health improved. However, the imperative to decide our actions by acting in the way in which we would like others to act would reverse the priorities of potential patients as research participants: health and knowledge gain are of wide social value. Professionals should, as a matter of duty, seek to learn by participation in implementation research, and contribute to knowledge for other professionals arising from the results of implementation research. This professional duty is also consistent with an Aristotlian ethic of living one's professional life well.

The dominance of informed consent in biomedical codes is consistent with a primary focus on autonomy. Comfortable, individualist societies that take the existence and reasonable quality of health care staff and facilities for granted can afford to focus on autonomy. Respect for autonomy is one principle in a popular scheme for medical ethics proposed by Beauchamp and Childress [34] that has four principles. The other three principles are non-maleficence (do no harm), beneficence (do good), and justice. If non-maleficence is only concerned with present patients, the harm caused by ignorance arising from the failure of implementation research will be treated as irrelevant. The benefit lost by preventing research through informed consent requirements will often be remote from the present patient. However, it is possible with chronic conditions that most of the people who will benefit from the results of particular research are those who are eligible to participate in that research. Unless 'justice' is interpreted to mean substantial responsibility towards other people, rather than a reasonable chance of getting access to goods, it will not be able to trump 'autonomy'. A proponent of autonomy would assert that autonomy, hence informed consent, is primary, and harm, loss of benefit, or extensive justice are less important.

Ordinary people can recognise that informed consent can raise a problem, and that their concerns about confidentiality might be addressed by other means [35]. The Canadian Institutes of Health Research and UK Medical Research Council guidelines recognize that consent might render epidemiological research impossible [24]. Aggregate data on populations of patients respects privacy, so perhaps implementation research that uses aggregate data should not seek consent from individual patients. The UK does not require consent [25] for the use of such aggregate data. However, with aggregate data, patient level effects cannot be modeled: analysis at the cluster level removes a potentially important level of explanation and understanding.

#### Social science codes

The Nuremberg Code and the Declaration of Helsinki aim to protect patients: but they have limited relevance when subjects are health professionals, and the division of responsibilities between professionals, patients, and researchers must be considered. In contrast to the four principles of Beauchamp and Childress, obligations to four groups of people form the framework used by the International Statistical Institute Declaration on Professional Ethics [13], from which most social science codes are derived [36-40].

The groups to whom obligations are owed by statisticians (taken as an example of social scientists) are: society, funders and employers, colleagues, and subjects. No one group has priority. Social obligations require statisticians to consider conflicting interests and guard against misuse and misinterpretation of statistics, to benefit as large a community as possible, and to pursue reasonable objectivity. Hence the interests of patients, doctors, and managers in promoting the uptake of clinical research must all be recognised, and the study design must facilitate interpretation and generalisation of results. Obligations to funders or employers require clarity about roles and responsibilities; transparency of statistical methods; no pre-emption of outcomes; and safeguarding privileged information. For example, if a funder of implementation research insisted on informed consent, a statistician may have to state that she cannot ensure that there will be data relevant to the funder's chosen outcome, as well as what limitations arise from data on a biased subset of people.

## Summary

Greater access to well-grounded information benefits society, so implementation research, which endeavours to translate improvements in clinical research into improvements in health care, is ethically commendable. Implementation research should be guided more by the principles of social science research, with the clinical treatment of patients being governed by ordinary professional practice. Seeking individual informed consent is not merely expensive: it may be futile, as those choosing to respond will almost certainly be unrepresentative, hence the study results will be biased. In reality, societies may face a political decision between individual informed consent and implementation research.

While ethical justification for clinical trials relies heavily on individual consent, for implementation research aspects of distributive justice, economics, and political philosophy underlie the debate. These ethical issues have been thoroughly debated in the social sciences. Biomedical codes focus on doctor-patient relations, whereas obligations to a variety of interest groups, ownership of information, rights of access and exploitation of data, and responsibilities for professional development are addressed in social science codes. We suggest that social sciences codes could usefully inform the consideration of implementation research by members of research ethics committees. We recommend that training on the particular features of implementation research be offered to those on research ethics committees.

## Competing interests

JLH: none declared. MPE and JMG have both submitted implementation trial protocols to ethics committees and had difficulty explaining to them the differences between implementation trials and individual patient clinical trials.

## Authors' contributions

JLH, ME and JMG together developed the idea for this paper. JLH led the writing. All authors commented on sequential drafts and approved the final version.
